# Discovery of a novel HDACi structure that inhibits the proliferation of ovarian cancer cells *in vivo* and *in vitro*

**DOI:** 10.7150/ijbs.62339

**Published:** 2021-08-12

**Authors:** Miao Bai, Mengqi Cui, Mingyue Li, Xinlei Yao, Yulun Wu, Lihua Zheng, Luguo Sun, Qiuhang Song, Shuyue Wang, Lei Liu, Chunlei Yu, Yanxin Huang

**Affiliations:** 1 National Engineering Laboratory for Druggable Gene and Protein Screening, Northeast Normal University, Changchun, 130024, China.; 2 Research Center of Agriculture and Medicine gene Engineering of Ministry of Education, Northeast Normal University, Changchun, 130024, China.; 3 Hebei Key Laboratory of Chinese Medicine Research on Cardio-Cerebrovascular Disease, Hebei University of Chinese Medicine, Shijiazhuang, China.

**Keywords:** HDACi, EOC, c-Myc, HDAC7

## Abstract

Histone deacetylases (HDACs) exhibit increased expression in cancer and promote oncogenesis via the acetylation of or interactions with key transcriptional regulators. HDAC inhibitors (HDACis) decrease HDAC activity to selectively inhibit the occurrence and development of tumors. Our study screened and obtained a new HDACi structure. *In vitro* experiments have showed that among the leads, Z31216525 significantly inhibited the proliferation and induced the apoptosis of epithelial ovarian cancer (EOC) cells. *In vivo* experiments demonstrated that compared to the control, Z31216525 significantly inhibited tumor growth and showed very low toxicity. Further mechanistic studies revealed that Z31216525 may exert an antitumor effect by inhibiting the expression of the c-Myc gene. Collectively, our studies identified a novel HDACi that is expected to become a new potential therapeutic drug for EOC and has important value for the design of new HDACi structures.

## Introduction

Histone deacetylases (HDACs) and histone acetyltransferases (HATs) 1 regulate gene expression via chromatin modification 2. Under normal physiological conditions, HDACs and HATs regulate histone acetylation in a balanced manner. When the cell undergoes transformation, HDAC activity is significantly enhanced 3 and disrupts the original balance of gene expression, leading to an imbalance of oncogenes that affect cell proliferation and regulate cell cycle progression, which leads to the malignant transformation of cells 4. HDACs are divided into four groups according to the homology of yeast classification: type I HDACs (HDAC1/2/3/8), type IIa HDACs (HDAC4/5/7/9), type IIb HDACs (HDAC6/10), type III HDAC (Sir1-7) and type IV HDAC (HDAC11) 5. Class I, class II, which have been further divided into class IIa and class IIb, and class IV HDACs are Zn^+^-dependent metallohydrolases. Class III HDACs are NAD^+^-dependent enzymes 6. Many studies demonstrated that the abnormal expression of HDACs was related to a variety of tumors. For example, HDAC2 and HDAC3 are highly expressed in breast cancer 7, HDAC2 overexpression is negatively correlated with survival in hepatocellular carcinoma (HCC) 8, HDAC5 overexpression may promote the proliferation and differentiation of HCC cells by upregulating the expression of slx1 9, HDAC7 is overexpressed in colon cancer and chronic lymphoid leukemia (CLL) 10, and HDAC2 overexpression in gastric cancer is generally associated with tumor invasion 11.

HDAC inhibitors (HDACis) are a class of natural and synthetic compounds. Due to the different effects of each type of HDAC in cells, HDACis induce many cellular changes in cancer cells and suppress multiple pathways related to tumorigenesis. The U.S. Food and Drug Administration (FDA) has approved four HDACis, vorinostat, romidepsin, belinostat and panobinostat, for the treatment of cutaneous T cell lymphoma (CTCL) and peripheral T cell lymphoma (PTCL) 12-16 and recently approved a fifth HDACi, crustacean amide, for the treatment of PTCL in China 17. At least 15 kinds of HDACis are undergoing clinical research for single-drug or combination therapy for hematological malignancies and solid tumors 18. The discovered HDACis are approximately divided into four main categories based on their structure: hydroxamic acid, cyclic peptide, benzamide and short-chain fatty acid 19. Three of the four FDA-approved anticancer HDACis (vorinostat, belinostat and panobinostat) are hydroxamic acids 10, 20. HDACis induce cancer cell cycle arrest, differentiation and death. These compounds also inhibit angiogenesis and regulate immune responses 21. The different mechanisms of action of these compounds may depend on the type of cancer, the individual HDACi, dose, and other factors. For example, valproic acid (VPA) inhibits the invasiveness of bladder cancer cells, but not the invasiveness of prostate cancer cells 22. Although some progress has been made, the anticancer mechanisms of HDACis have not been fully elucidated. These drugs also have some adverse effects, including thrombocytopenia, neutropenia, nausea, vomiting, diarrhea, and fatigue 23-25. Therefore, an urgent need for HDACis with high efficiency, low toxicity and high selectivity remains. The anticancer mechanisms of HDACis also need further research 26.

In our research, we chose HDAC8 as the protein receptor for virtual screening. When studying the structure and functional characteristics of human HDACs, the active center of HDAC8 is the most extended type among a variety of HDACs. Therefore, it is compatible with a variety of different structures. The body has better adaptability when entering its active center 27-29, which makes HDAC8 a good choice for computer-assisted drug screening. We identified 14 novel HDACis using a virtual screening process, performed activity tests, and ultimately discovered the Z31216525 compound, which significantly inhibited HDAC activity. It is a new structure of HDACi that produced strong chelation with Zn in the active center of HDACs. Further *in vitro* experiments showed that Z31216525 selectively inhibited the proliferation of cancer cells and had little effect on normal cells. To explore the mechanism of Z31216525 inhibition of ovarian cancer cell proliferations, we used RNA sequencing (RNA-seq) to identify differentially expressed genes. Protein-protein interaction (PPI) network analysis suggested that the activity of c-Myc played a key role in the antitumor effect of Z31216525. The experimental results showed that upregulation of the c-Myc gene significantly reversed the antiproliferative effect of Z31216525 on ovarian cancer cells. Further *in vivo* experiments showed that Z31216525 significantly decreased the viability of ovarian cancer cells and very low toxicity in nude mice. Our findings improved our understanding of the antitumor mechanism of HDACis, and indicate that Z31216525 is a new type of HDACi with potential as a treatment for ovarian cancer.

## Materials and methods

### Antibodies and reagents

Antibodies and reagents used in the study are listed as follows: anti- Cleaved Caspase3 was purchased from Cell Signaling Technology (1:1000) (Boston, America) Anti-Histone H3 (acetyl K27) (ab4729, 1:2000), anti-Cyclin D2 (ab230883, 1:2000) were purchased from Abcam company (Cambridge, England). Anti-c-Myc (10828-1-AP, 1:1000), anti-CDK4 (66950-1-Ig, 1:1000), anti-BOP1 (28366-1-AP, 1:1000), anti-NOP56 (18181-1-AP, 1:1000), anti-GAPDH (60004-1-Ig, 1:1000), anti-P21 (60214-1-Ig, 1:1000), anti-HDAC7 (26207-1-AP, 1:1000) and anti- Histone H3 (17168-1-AP, 1:1000) were purchased from Protein-tech Group (Wuhan, China). Dissolved Z31216525, Z46582199, Z165155756 and Z234820564 (Enamine Ltd, Ukraine) in DMSO separately at a concentration of 10 mM.

### Pharmacophore screening and molecular docking

We followed the screening method of the previous article 30. The Enamine (https://enamine.net/) database was screened using the pharmacophore screening module of SYBYL X2.0 (http://www.tripos.com). Then, we used GOLD5.2 (http://www.ccdc.cam.ac.uk) to perform molecular docking screening on the screening results. The interaction analysis between drug molecules and target proteins used Ligplot (https://www.ebi.ac.uk/thornton-srv/software/LigPlus/) software. Molecular 3D structure analysis and drawing used Pymol (https://pymol.org/2/) software.

### Cell culture

Culturing A2780 (Human epithelial ovarian cancer), Skov3 (Human epithelial ovarian cancer), HepG2 (Human liver cancer), Bel7402 (Human liver cancer), SGC7901 (Human stomach cancer), HeLa (Human cervical cancer), SW480 (Human colon cancer), HOSEpiC (Human ovary) and LO2 (Human liver) cell lines were obtained from the cell library of the Chinese Academy of Sciences. All cell lines were cultured in 1640 medium with 10% FBS (TBD, China) and incubated at 37 °C in an incubator with 5% CO_2_.

### Evaluation of HDAC enzyme activity *in vitro*

We used the HDACi screening kit (BioVision, K340, USA) to screen lead compounds with HDAC inhibition. Experimental method reference literature 30. Enzyme activity ratio calculation method: (OD value of test HDACi/OD value of water) × 100%. The data was displayed by the means of mean ± SE.

### DAPI staining

DAPI staining was used to counterstain the nuclei or to observe apoptotic bodies. Cultured cells were washed twice with PBS and then fixed with 4 % paraformaldehyde for 15 min at 37 °C. Then, the cells were stained with DAPI 1:10 000 for 10-15 min in the dark after being washed with PBS. Apoptotic bodies were observed using a fluorescence microscope.

### Cell proliferation assay

We used MTT assay and BrdU incorporation to analyze cell proliferation. The MTT assay was analyzed under wavelength of 490nm to read the absorbance of each well. The calculation method of the inhibition rate was as follows: OD of experimental group/ (OD of control group-OD of blank group) × 100% 31. The data was presented as means ± SE. The BrdU incorporation was analyzed under the wavelength of 450nm to read the absorbance value of each well. The calculation method of the inhibition rate was (OD value of test group/OD value of control) × 100%.

### Apoptosis and cycle analysis

To analyze apoptosis and cell cycle, we used Z31216525 to incubate with cells for 0 h, 12 h, 24 h, 36 h, 48 h in A2780 and Skov3 cells. In cell cycle analysis, we fixed the collected cells in 75% alcohol at 4°C for 24 h, then incubating with propidium iodide staining solution at 37 °C for 30 min and then using flow cytometry to detect. The analysis software selected Modfit (Verity Software House, Topsham). In apoptosis detection, we incubated the collected cells with propidium iodide staining solution for 15 min and used flow cytometry to detect apoptosis ratio.

### Clone formation detection

We took log-phase A2780 and Skov3 cells and treated them with trypsin, cultured about 300 cells in each well of a six-well plate, added 60 mM Z31216525 or an equivalent amount of DMSO after 24 h, and cultured them for about 1 to 2 weeks. Washed with PBS three times, and then fixed with 4% paraformaldehyde for 30 min at room temperature, dyed with 2.5% crystal violet, and placed at room temperature for 10 min. Washing the six-well plate with PBS three times and air-dry, taking pictures and count. We repeated the experiment three times each time.

### RNA sequencing analysis

A2780 cells were incubated with DMSO or Z31216525 for 0 h, 4 h, 16 h, 24 h, and each group of cells in three independent replicates. The mRNA was extracted and the total mRNA was quantified. The RIN of the total mRNA of each sample was 10, and the A260/280 ratio exceeded 2.1. BGI (Hong Kong) used the BGISEQ-500 sequencer to perform transcriptome analysis on RNA-Seq. We have uploaded the sequencing data to the SRA public database (PRJNA727789), ID: SUB9578775. In cluster analysis, correlation heat map, pathway analysis and GO analysis, we all used BGI's multi-omics system platform for analysis. We used the combination of STRING website 32 (https://www.string-db.org/) and Cytoscape software 33 (https://cytoscape.org/) to construct protein network interaction diagrams.

### Real-time quantitative PCR

Total RNA was extracted with TRIzol reagent, and then reversing transcribed into cDNA using a real-time PCR kit (TransGen Biotech, Beijing, China). RT-qPCR was performed using SYBR Green I PCR Master Mix Kit (TAKARA, Beijing, China). The following sequences of the gene-specific primer pairs were used as Table S1.

### Western blot

We had prepared whole cell lysates, cytoplasmic and nuclear protein lysates to Western blotting. The primary antibody (Proteintech Group, Abcam) was incubated at 4 °C for 12 h, and the secondary antibody (Proteintech Group) was incubated at room temperature for 2 h. GAPDH and H3 were used as controls.

### RNA interference and overexpression

The interference plasmid and negative control plasmid of c-Myc, the overexpression plasmid and negative control plasmid of pEGFP-c-Myc were purchased from Sagon Biotech (Shanghai, China). The shRNA sequences for c-Myc-knockdown were as follows: (AAACAACAUCGAUUUCUUCCU GAAGAAAUCGAUGUUGUUUCU). Cells were seeded at 50 % confluency into 6-well plates before transfection. Then shRNAs were transfected into the cells using lipofectamine 2000 reagent following the manufacturer's instructions.

### Animal experiment

The animal experiment protocol had been approved by the Ethics Committee of Northeast Normal University. Twelve nude female mice (about 4-week-old) were purchased from the SLAC Laboratory, Shanghai, China. The tumor xenografts were induced by subcutaneously inoculating A2780 cells (1 × 10^6^/100 uL) into the right flank region. Tumor size and body weight were measured at last, and tumor xenograft volume was calculated using the following formula: V = ab^2^/2 (a: the long diameter and b: the short diameter). The tumor xenografs were isolated at the endpoint of experiment, and the tumor size and weight were compared by using the statistical analysis.

### Statistical analysis

The data were processed using one-way ANOVA with SPSS software (SPSS Inc., Chicago, USA), and all data shown are presented as the mean ± SE of at least three independent experiments. ***p < 0.001, **p < 0.01, and *p < 0.05 were considered statistically significant.

## Results

### Virtual screening of HDACis

In our previous research 30, a screening process was designed based on HDACis and the Enamine database to obtain potential HDACis. After analysis, 14 candidate compounds with different structures were obtained for further research.

### Evaluation of the inhibition of enzyme activity and analysis of the docking results

**Figure 1:**
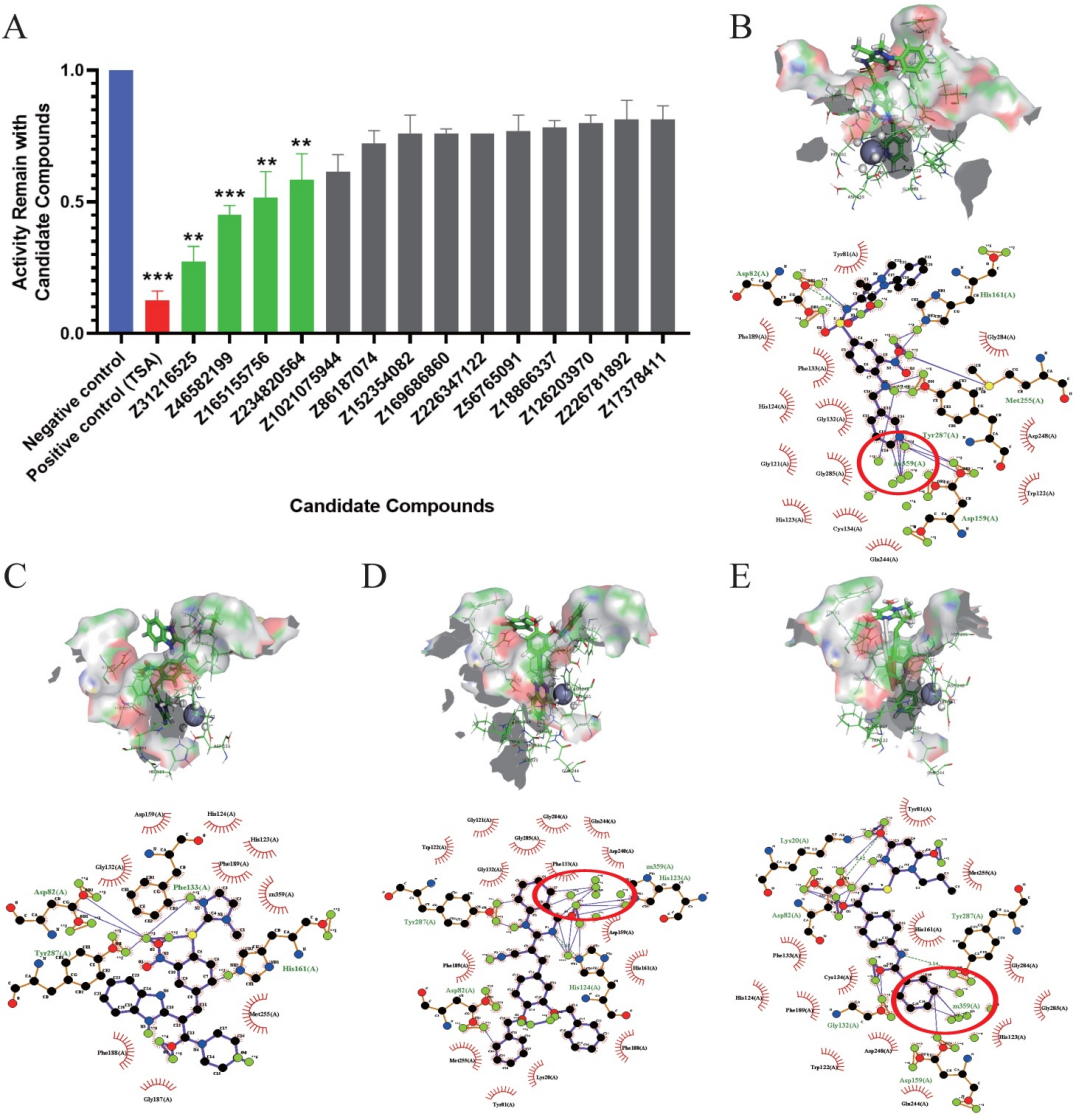
** Evaluation of the inhibition of enzyme activity and analysis of the docking results. (A)** Inhibition rates of HDAC enzyme activity of the 14 docking hit compounds. The positive control is shown in blue, the inhibitor control is shown in red, and hit compounds are shown in green. The result is the average of three independent experiments, and t-tests were performed with the Negative control as the standard, *p <0.05; **p<0.01; ***p<0.001. **(B-E)** Pose diagram and force analysis of the docking between Z31216525, Z46582199, Z165155756 and Z234820564 and the active center of the HDACis.

The inhibitory effects of 14 candidate compounds on HDACs were detected using a fluorescent HDAC activity kit. We chose trichostatin A (TSA) as a positive control, because TSA is a classical and widely used HDACi 34. The results showed that four compounds Z31216525, Z46582199, Z165155756 and Z234820564 significantly inhibited HDAC activity, and the structures of these four compounds are shown in Fig S1. Treatment with candidate compounds reduced HDAC activities to 27. 44%, 45.00%, 51.50% and 58.45%, respectively. However, the other 10 candidate compounds had little inhibitory effect (Fig. 1A).

To further analyze the binding mode of the compounds and the active center of HDAC, we used ligplot to analyze the docking postures with the highest scores when the four compounds were docked with HDAC8. Z31216525 formed hydrogen bonds with the amino acids Asp82, Asp159, His161, Met255 and Tyr287 in the active center region (Fig. 1B), Z46582199 formed hydrogen bonds with the amino acids Asp82, Phe133, His161, and Tyr287 in the active center region (Fig. 1C), Z165155756 formed hydrogen bonds with the amino acids Asp82, His123, His124 and Try287 in the active center region (Fig 1D), and Z234820564 formed hydrogen bonds with Lys20, Asp82, Gly132, Asp159 and Tyr287 in the active center region (Fig. 1E). The combinations show that the four compounds have a large number of hydrogen bonds with the active center, and they all bound well to HDAC8.

The formation of the chelate ring makes the chelate more stable than the nonchelating complex with a similar composition and structure. Among the four compounds, Z31216525, Z165155756, and Z234820564 chelated Zn in the active center 6. C7 and C1 in the benzene ring of Z165155756 chelated Zn in the active center (Fig. 1D), C19 and C20 in the benzamide functional group of Z234820564 chelated Zn (Fig. 1E). Compared to these two compounds, Z31216525 had a unique structure. C14, C15 and N4 in the pyridine functional group formed a strong chelating effect with Zn in the active center (Fig. 1B), and the number of chelating rings formed was the largest. Therefore, it had the strongest binding effect with HDAC8.

### Anticancer action mechanism of candidate compounds

**Figure 2:**
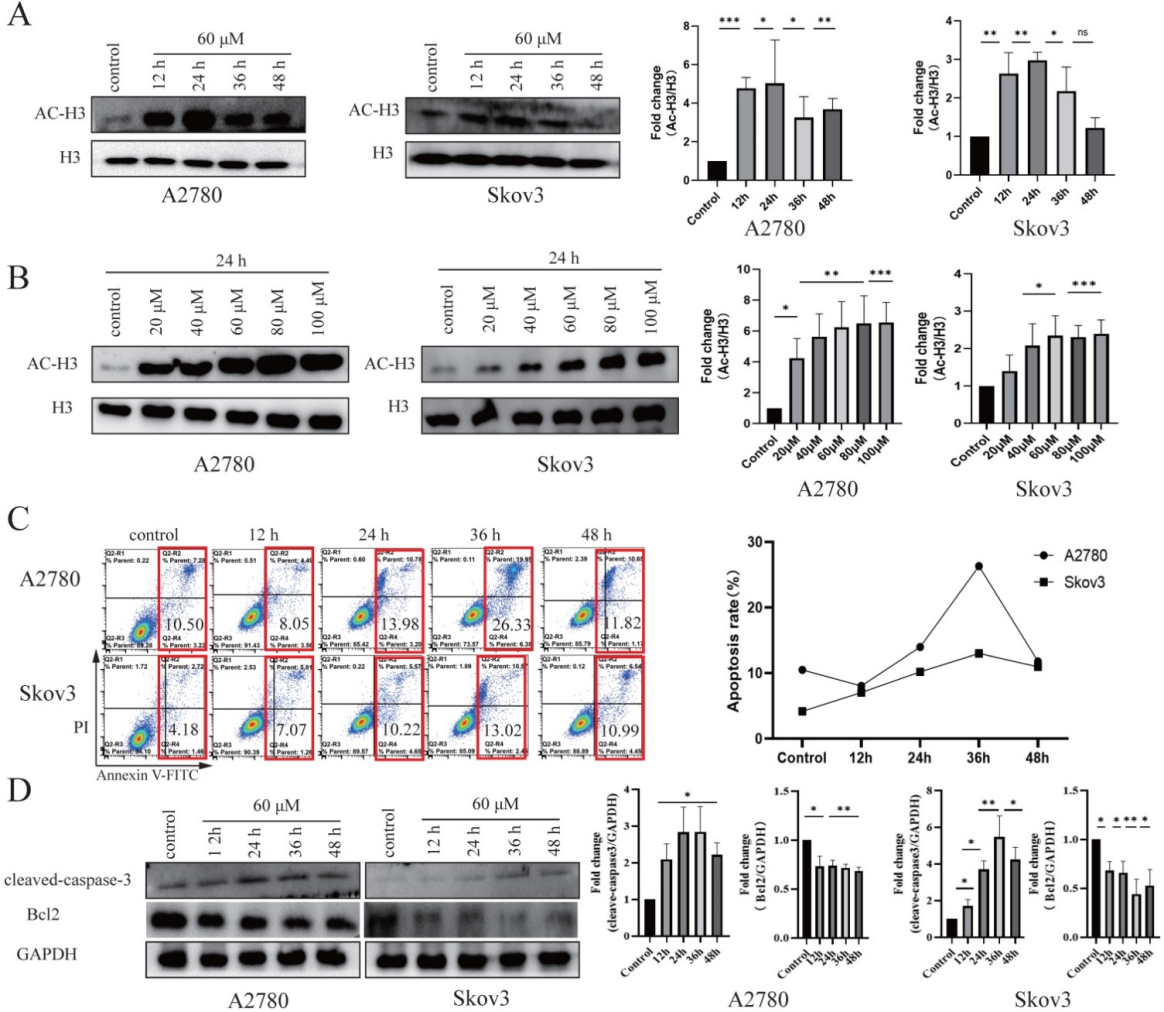
** Z31216525 promotes apoptosis in A2780 and Skov3 cells via the mitochondrial pathway. (A)** The effects of Z31216525 treatment for different times on acetylated histone H3, with H3 as a control, in A2780 and Skov3 cells. **(B)** The effects of Z31216525 treatment for different concentrations on acetylated histone H3, with H3 as a control, in A2780 and Skov3 cells. **(C)** DMSO control and Z31216525 treatment for 12 h, 24 h, 36 h, and 48 h. Cell apoptosis percentage. **(D)** Cells were treated with 60 μM Z31216525 for 12 h, 24 h, 36 h, and 48 h, and cells were treated with an equal volume of DMSO for 48 h as a control. Western blotting analysis was used to determine the protein levels. These experiments are representative of at least three independent experiments, and t-tests were performed, *p <0.05; **p<0.01; ***p<0.001.

HDACis specifically induce cell cycle arrest and apoptosis 35, 36. To test the effects of these four drug candidates, cell viability was detected using the MTT assay in seven cancer cell lines (A2780, Skov3, HepG2, Bel7402, SGC7901, HeLa and SW480) and a normal cell line (HOSEpiC). After treatment with Z31216525, Z46582199, Z165155756 and Z234820564 for 48 h, the IC50 values of these four drugs in cancer cell lines and the IC10 value in the HOSEpiC cell line were analyzed (Table 1). Z31216525 had the strongest inhibitory effect. The IC50 values of Z31216525 on A2780, Skov3, HepG2, Bel7402, HeLa, SW480 and SGC7901 cells were 59.98±7.40, 64.12±5.22, 68.52±5.28, 59.42±4.18, 87.52±5.7, 112±22.2, and 153.739±16 μM, respectively. The IC10 value was 87.55±3.24 μM in HOSEpiC cells. The IC10 value in HOSEpiC normal ovarian cells were greater than the A2780 and Skov3 ovarian cancer cells. These results showed that Z31216525 exerted antitumor effects against all tumor cell lines, especially ovarian cancer cell lines. Western blotting results demonstrated that Z31216525 significantly increase the acetylation level in EOC cells at 12 h, 24 h, 36 h, and 48 h, especially at 24 h (Fig. 2A), and the acetylation level in EOC cells was also significantly up-regulated as the concentration increases (Fig. 2B).

According to the IC50 values of Z31216525 for A2780 and Skov3 cells and the IC10 value for HOSEpiC cells, 60 μM was selected as the optimal concentration for subsequent experiments.

We tested the effects of Z31216525 on ovarian cancer cell apoptosis. DAPI staining showed that Z31216525 induced the formation of apoptotic bodies and apoptotic cells in A2780 and Skov3 cells (Fig. S2). The two epithelial ovarian cancer cell lines were treated with Z31216525 for 0 h, 12 h, 24 h, 36 h, and 48 h, and flowcytometry revealed that the apoptosis rates in A2780 cells were 10.50%, 8.05%, 13.98%, 26.33%, and 11.82%, respectively. The apoptosis rates of Skov3 cells were 4.18%, 7.07%, 10.22%, 13.02%, and 10.99%, respectively, after Z31216525 treatment (Fig. 2C). Western blotting results showed that the protein expression of cleaved caspase3 was upregulated in A2780 and Skov3 cells (Fig. 2D). The activation of caspase3 induces cell apoptosis 37. Z31216525 also downregulated the expression of Bcl-2. Bcl2 and cleaved caspase-3 are mitochondria-associated proteins. The present study suggested that Z31216525 induced the apoptosis of A2780 and Skov3 cells via the mitochondrial pathway, and the apoptosis rate had a close relationship with the treatment time.

**Figure 3:**
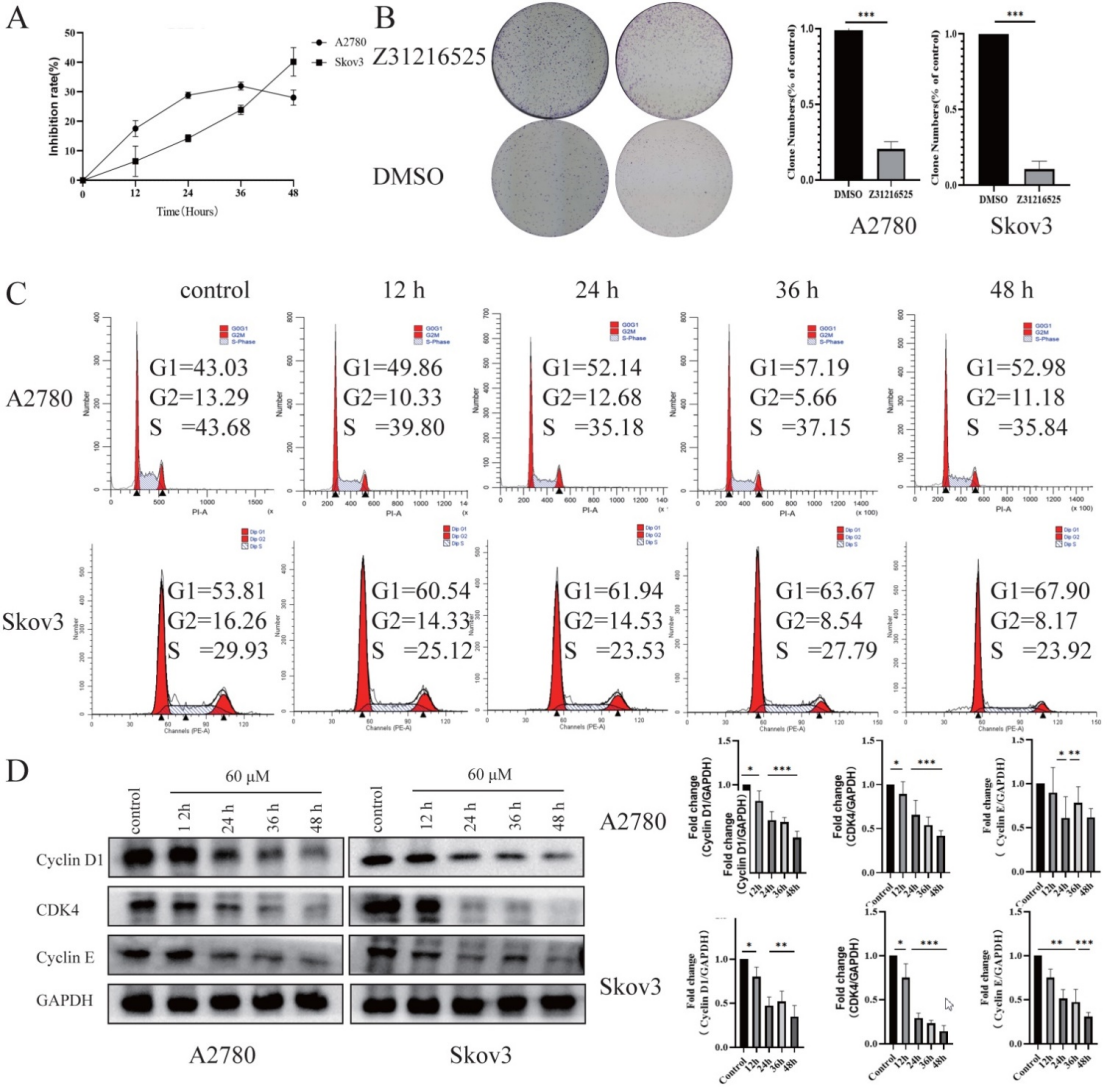
** Z31216525 induced cell cycle arrest in A2780 and Skov3 cells. (A)** A2780 and Skov3 cells were treated with Z31216525 (60 μM) for 12 h, 24 h, 36 h, and 48 h. The BrdU incorporation method was used to analyze the antiproliferative effect of Z31216525 on cells. **(B)** The effect diagram of the colony formation experiment. **(C)** DMSO control and Z31216525 treatment for 12 h, 24 h, 36 h, and 48 h. Cell cycle distribution diagram. **(D)** Cells were treated with 60 μM Z31216525 for 12 h, 24 h, 36 h, and 48 h, and cells were treated with an equal volume of DMSO for 48 h as a control. Western blotting analysis was used to determine the protein levels. These experiments are representative of at least three independent experiments, and t-tests were performed, *p <0.05; **p<0.01; ***p<0.001.

We tested the effects of Z31216525 on the proliferation of ovarian cancer cells. The results of BrdU and colony formation experiments showed that Z31216525 significantly inhibited the proliferation of A2780 and Skov3 cells (Fig. 3A-B). We analyzed the cell cycle changes after treatment with Z31216525 using flow cytometry. After Z31216525 treatment for 12 h, 24 h, 36 h and 48 h, the proportions of A2780 cells in the G1 phase increased 6.83%, 9.11%, 14.16%, and 9.95%, respectively, compared to the control treatment. The proportion of Skov3 cells in the G1 phase increased 6.97%, 8.13%, 9.86%, and 14.09%, respectively (Fig. 3C), and the results showed that Z31216525 induced G1 phase arrest in the two kinds of cells. Cyclin D, cyclin E and CDK4 play important roles in the transition from G1 to S phase of the cell cycle 38. Therefore, we further performed Western blotting to analyze the expression of these three proteins. As shown in the figure, treatment with Z31216525 significantly downregulated the protein expression of cyclin D, cyclin E and CDK4 in A2780 and Skov3 cells (Fig. 3D). These data suggest that Z31216525 inhibits the proliferation of ovarian cancer cells by inducing G1 phase arrest.

Briefly, Z31216525 induced the apoptosis of upper ovarian epithelial cells via the mitochondrial pathway, but the level of apoptosis was not remarkable. Flowing cytometry, BrdU and colony formation experiments showed that Z31216525 blocked EOC cells in the G1 phase and significantly inhibited cell proliferation. In summary, Z31216525 exerted an antitumor effect primarily via inhibition of EOC cells proliferation.

### Analysis of sequencing results after Z31216525 treatment of A2780 cells

**Figure 4:**
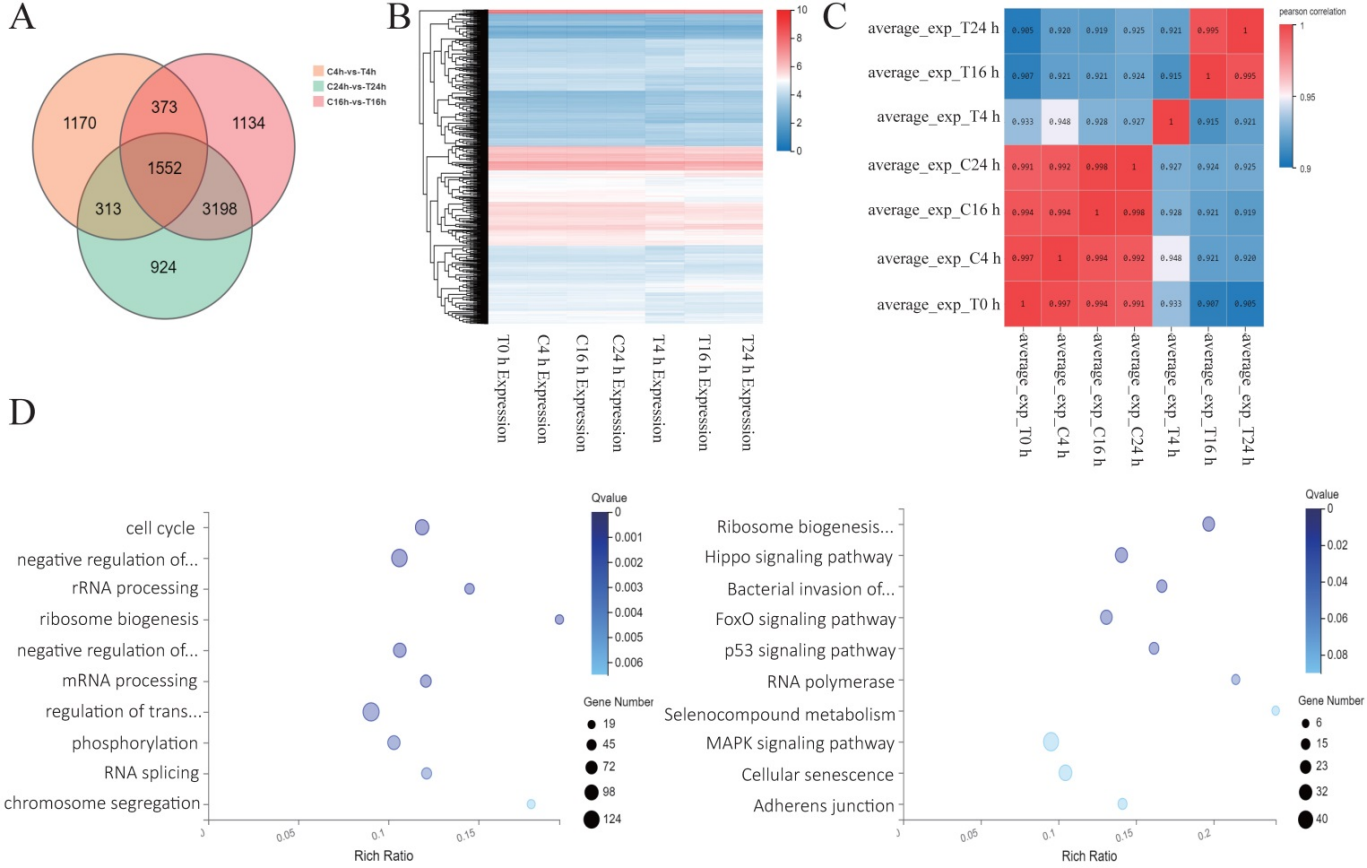
** Analysis of sequencing results after Z31216525 treatment of A2780 cells. (A)** Analysis of the differentially expressed genes in A2780 cells 4 h, 16 h, and 24 h after treatment with Z31216525. **(B-C)** Heat maps were drawn according to the clustering of differentially expressed genes at three time points. **(D)** The pathway and GO analysis of differentially expressed genes overlapping among all three time points.

To further explore the antitumor mechanisms of Z31216525, we treated A2780 cells with DMSO and Z31216525 for 0 h, 4 h, 16 h and 24 h. Each group included three independent replicates, and RNA-seq was used to comprehensively analyze the changes in the mRNA expression. The numbers of differentially expressed genes at 4 h, 16 h and 24 h were 3408, 6257 and 5987, respectively. A total of 1552 differentially expressed genes between the control group and treatment group overlapped among the three time points (Fig. 4A). We also performed a cluster analysis of the genes with significant differences at the three time points. These genes clustered well in the control group and the treated group and had good reproducibility (Fig. 4B). The correlation heat map showed that there were basically no significant changes at the four time points for the control group. Gene expression began to change in the drug-treated group after 4 h of treatment. Gene expression remained basically unchanged from the 4-h point to the 16-h and 24-h time points, and gradually stabilized (Fig. 4C). We performed GO analysis and pathway analysis at three time points and found that the differentially expressed genes were primarily related to the cell cycle and ribosomal biogenesis. The differentially expressed genes were primarily enriched in ribosomal biogenesis pathways and cancer-related pathways (Fig. 4D).

**Figure 5:**
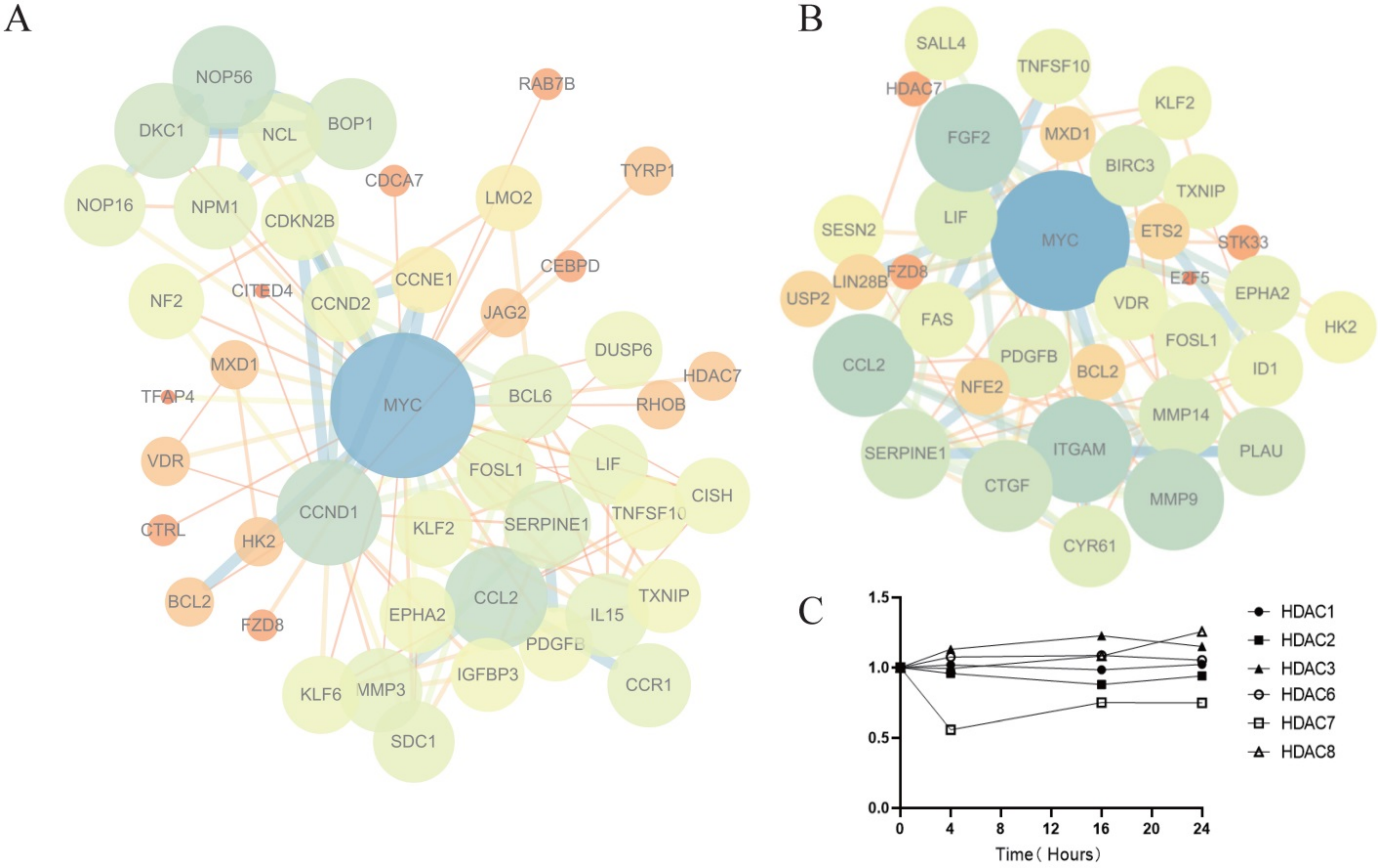
** Analysis of key genes involved in the antitumor effect of Z31216525. (A)** The PPI network map for 4 h, 16 h and 24 h (|FC| >0.5, Q value<0.05). **(B)** The PPI network map for 4 h (|FC| >1, Q value<0.05). **(C)** Changes in HDAC mRNA expression (treatment group/control group) after Z31216525 treatment for 4 h, 16 h and 24 h.

We constructed a PPI network map for genes with significant differences at three time points and found that the c-Myc gene was in the center of the network (Fig. 5A). The c-Myc gene is a key molecule in the regulation of cell processes, and the expression level of c-Myc is closely related to cell proliferation 39. Downregulation or inactivation of c-Myc gene expression damages the cell cycle process 40. The c-Myc gene activates the transcription of cell cycle-promoting genes such as Cdc25a, CDK4, cyclin D and cyclin E. c-Myc also inhibits the expression of cell cycle growth restriction genes, such as p15 and p21, to significantly promote cell cycle progression 41, 42. The expression of these genes was also shown in the sequencing results. c-Myc directly affects the biogenesis of ribosomes 43, 44. The sequencing results showed that marker genes of ribosomal biogenesis that are related to c-Myc, such as NOP56, NCL, BOP1, DCK1 and other genes, were significantly downregulated. We speculate that c-Myc plays a key role in the antitumor effect of Z31216525.

HDACis began to work at 4 h. To further explore the mechanism of Z31216525 downregulation of the c-Myc gene, we constructed a PPI network map for genes with significant differences at 4 h (Fig. 5B). The results showed that the epigenetic regulatory factor SALL4 was significantly downregulated after treatment with Z31216525 (FC=-0.84). SALL4 is a cancer marker that is abnormally expressed in ovarian cancer 45. c-Myc is the downstream target of SALL4. SALL4 binds to the promoter region of c-Myc to directly regulate its expression. Downregulation of SALL4 leads to decreased expression of c-Myc at the protein and mRNA levels 46, 47. Z31216525 triggers the activation of TRAIL by changing the activation of transcription factors, which results in the loss of c-Myc activity 48. MXD1 (FC=1.01) interacts with MAX to inhibit c-Myc transcription 49. The sequencing results showed that only HDAC7 mRNA expression was significantly downregulated (FC=-0.88) at three time points in A2780 cells treated with Z31216525 (Fig. 5C). Some HDACis, such as vorinostat, selectively downregulate HDAC7 and are associated with a reduction in HDAC7 mRNA levels 50. Therefore, Z31216525 may significantly inhibit HDAC7 at the mRNA and protein levels. HDAC7 directly binds to the c-Myc gene, and HDAC7 silencing decreases c-Myc mRNA levels by reducing histone H3/H4 acetylation and suppressing the association of RNA polymerase II (RNAP II) with the c-Myc gene 51. In conclusion, the downregulation of the key gene c-Myc is caused by the combined action of various regulatory factors.

### Differential gene pathway analysis and sequencing verification

**Figure 6:**
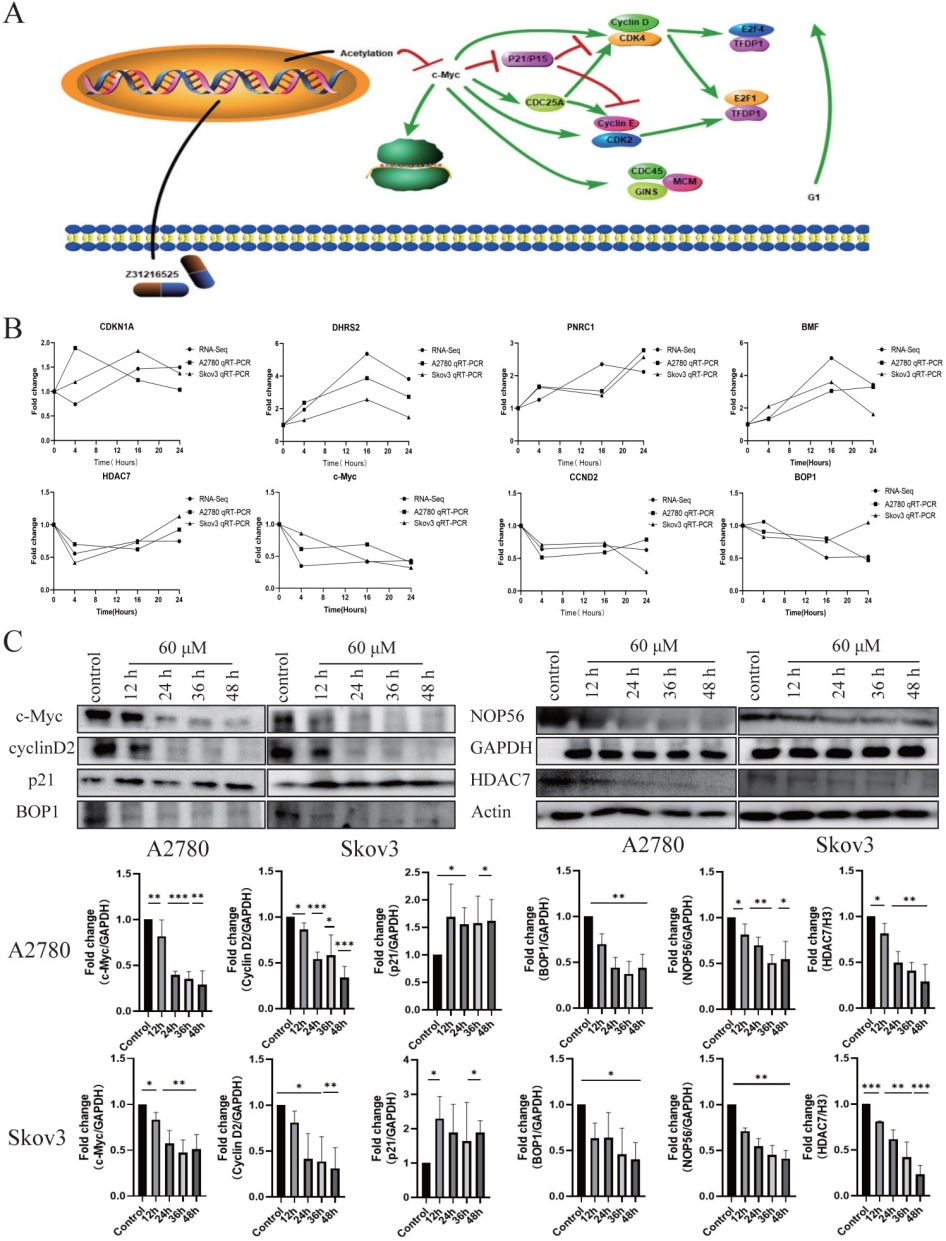
** Differential gene pathway analysis and sequencing verification. (A)** Differentially expressed gene pathway network constructed based on the cell cycle and ribosomal biogenesis pathways. **(B)** The qPCR experiment verified the changes in the mRNA expression of some genes after Z31216525 treatment at three time points in A2780 and Skov3 cells. The values are shown as the means ± SE of three independent experiments. **(C)** The expression of c-Myc-related proteins was verified at the protein level using Western blotting of samples from A2780 and Skov3 cells treated with Z31216525 for 12 h, 24 h, 36 h, and 48 h. These results are representative of at least three independent repetitions, and t-tests were performed, *p <0.05; **p<0.01; ***p<0.001.

Based on the above analysis, we proposed the potential mechanism of the antitumor action of Z31216525. Z31216525 acts, primarily by inhibiting the expression of HDAC7 and acetylation to inhibit the expression of c-Myc. The decrease in c-Myc gene expression affects the cell cycle pathway and the ribosome biogenesis pathway to, play an antitumor role (Fig. 6A). To verify the RNA-seq results and our pathway hypothesis, we used qPCR technology to confirm the expression of HDAC7, c-Myc, CCND2, BOP1, DHRS2, PNRC1, CDKN1A, and BMF (Fig. 6B). We also used Western blotting to verify that the changes in the expression levels of HDAC7, c-Myc, P21, cyclin D2, BOP1, and NOP56 at the protein level were consistent with the sequencing and qPCR results (Fig. 6C).

### c-Myc participates in the anticancer effect of Z31216525

**Figure 7:**
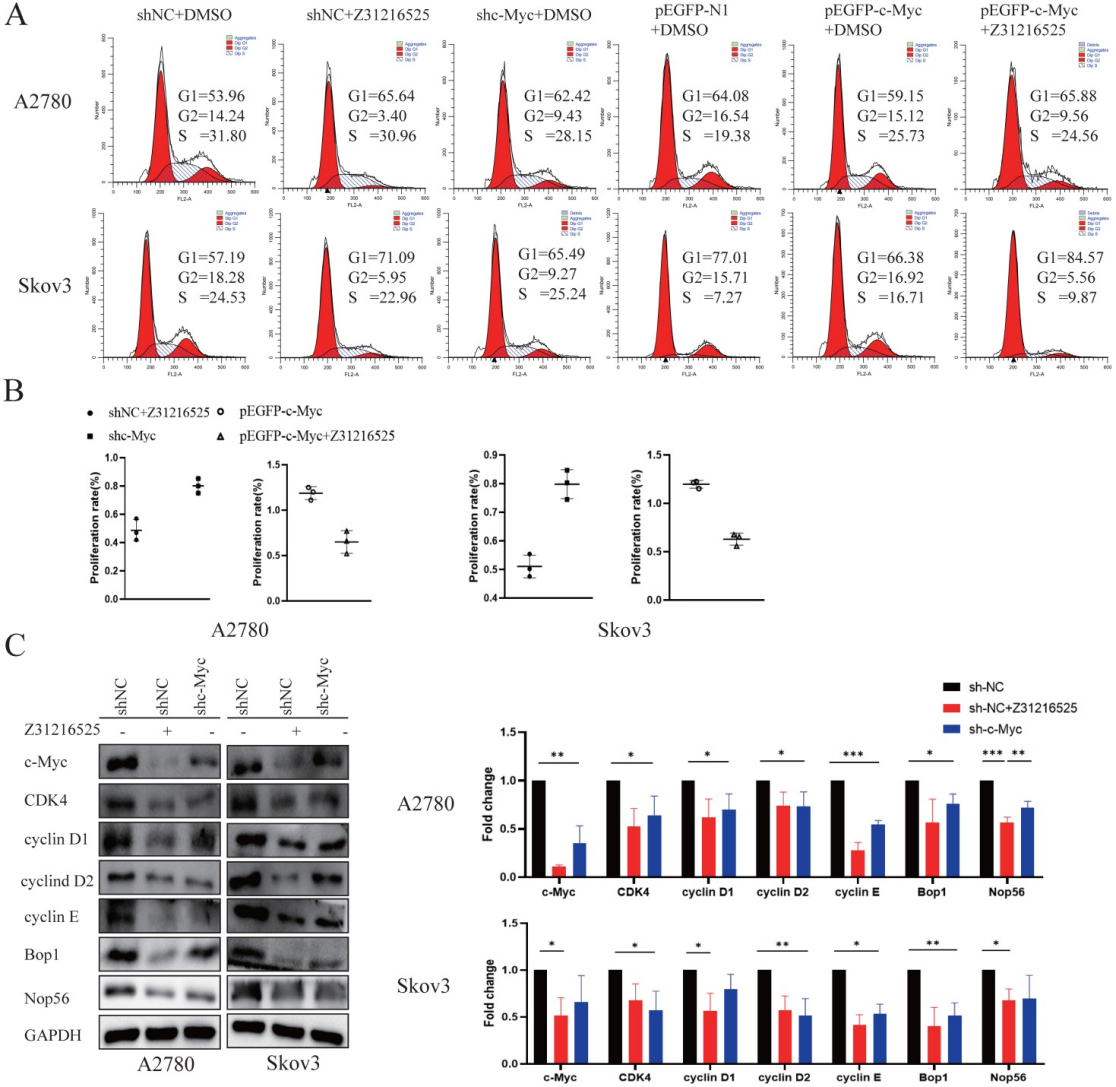
** c-Myc is involved in the anticancer effect of Z31216525. (A)** After 48 h of RNA interference, the cells were treated with DMSO or Z31216525 for 48 h then stained with propidium iodide to assess the cell cycle distribution using flow cytometry. **(B)** After 48 h of RNA interference, the cells were treated with DMSO or Z31216525 for 48 h, and the effects on cell proliferation were detected using a BrdU incorporation assay. **(C)** The cells were transfected with shRNA for 48 h then treated with Z31216525 or DMSO for 48 h, and the expression of c-Myc-related proteins was analyzed using Western blotting. These results are representative of at least three independent repetitions, and t-tests were performed, *p <0.05; **p<0.01; ***p<0.001.

To investigate whether the c-Myc gene was involved in cell proliferation inhibition induced by Z31216525, we constructed c-Myc short-hairpin RNAs (shRNAs) and expression plasmids (pEGFP-c-Myc), and the efficiency was measured using Western blotting (Fig. S3). We transfected A2780 cells and Skov3 cells with negative control shRNA (shNC) or c-Myc shRNA (shc-Myc) then treated the cells with Z31216525. Flow cytometry analyses indicated that the downregulation of c-Myc and treatment with Z31216525 increased the percentage of A2780 and Skov3 cells in the G1 phase, and the upregulation was more obvious in the shNC-transfected cells treated with Z31216525 (Fig. 7A). The BrdU results showed that the growth of the other two groups was inhibited compared to the shNC group, and the growth of the Z31216525 treatment group was the most strongly inhibited (Fig. 7B). We also transfected A2780 and Skov3 cells with different expression plasmids, pEGFP-N1, pEGFP-c-Myc, and pEGFP-c-Myc, and treated the cells with Z31216525. Flow cytometry results showed that the percentage of cells in the S phase of the overexpression group was significantly increased compared to that in the NC group, and the percentage of cells in the G1 phase was decreased. However, after treatment with Z31216525, the percentage of cells in the G1 phase of the overexpression group increased significantly after treatment with Z31216525, and the percentage of cells in the S phase returned to baseline (Fig. 7A). The BrdU uptake assay showed that the upregulation of c-Myc significantly inhibited the antiproliferative effects of Z31216525 in A2780 and Skov3 cells (Fig. 7B). We further used Western blotting to assess the expression of cell cycle-related genes in DMSO- and Z31216525-treated shNC and shc-Myc cells. As expected, Z31216525-treated and c-Myc-shRNA -transfected cells exhibited downregulation of cyclin D1, cyclin D2, cyclin E, CDK4, BOP1 and NOP56 compared to the NC group (Fig. 7C).

These results indicate that c-Myc may be partially involved in Z31216525-mediated EOC cell cycle arrest.

### Z31216525 treatment showed an inhibitory effect on ovarian cancer tumor growth *in vivo*

To investigate whether Z31216525 inhibited the growth of tumor cells *in vivo*, we used A2780 cells for xenotransplantation. Two weeks later, nude mice bearing tumor xenografts were randomly divided into five groups and received the following treatments for three continuous weeks: the negative control (NC) group received intraperitoneal (i.p.) DMSO; the positive control group received SAHA (50 mg/kg/d, i.p); and the treatment groups received Z31216525 i.p. (low concentration 25 mg/kg/d, medium concentration 50 mg/kg/d, high concentration 100 mg/kg/d). After five weeks of feeding, the mice were sacrificed, and tumor growth was assessed.

**Figure 8:**
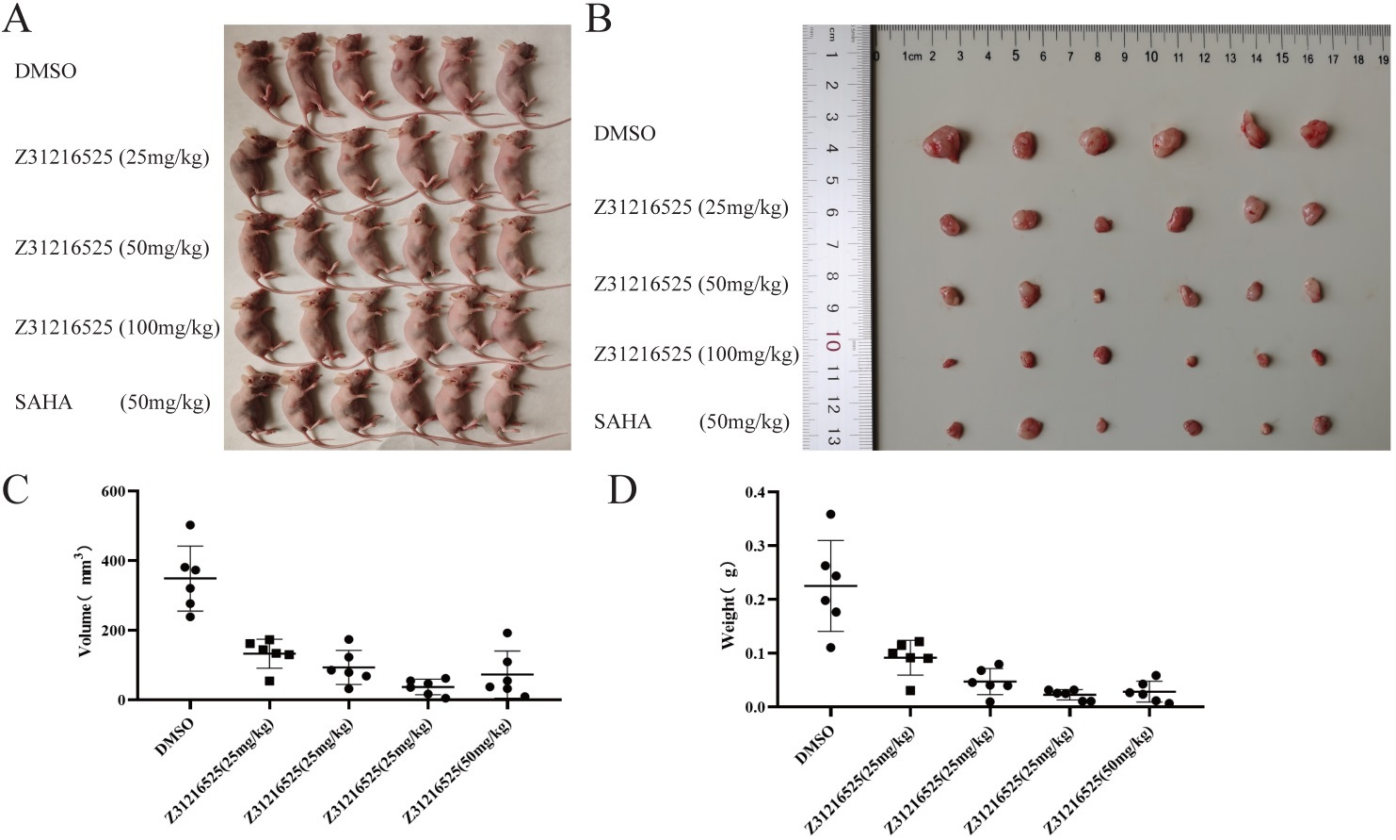
** Z31216525 treatment showed an inhibitory effect on ovarian cancer tumor growth *in vivo*. (A-B)** Mice and tumors.** (C-D)** The tumor sizes and weights of tumor-bearing mice were measured after 4- weeks of treatment.

Animals in the control group and the treatment group were healthy and normal, and there were no significant differences in appearance and weight (Fig. 8A, Table 2). The tumor volume and tumor weight were significantly reduced in the Z31216525 treatment group compared to the NC group (Fig. 8B-D), and the effects were dose-dependent. At the same concentration (50 mg/kg/d), the volume and weight of tumors in the Z31216525 group were slightly larger than the SAHA group. The volume and weight of tumors in the high-concentration Z31216525 (100 mg/kg/d) group were smaller than those of tumors in the SAHA group, and the treatment had no effect on the survival of the mice. In summary, Z31216525 significantly inhibited the growth of ovarian cancer tumors *in vivo* and showed very low toxicity.

## Discussion

HDACis have broad prospects in the treatment of cancer, and there are many reports about the potential for HDACi use in ovarian cancer. *In vitro* experiments showed that a variety of HDACis (valproic acid, sodium butyrate, vorinostat, belinostat, panobinostat and romidepsin) induced ovarian cancer cell cycle arrest and apoptosis 52-54. Vorinostat in combination with paclitaxel significantly increased survival in the intraperitoneal EOC model 55. The survival time of Panobinostat-treated mice in an orthotopic EOC xenograft model was significantly prolonged 56. Although the use of HDACis alone and in combination with other anticancer drugs completely changed the treatment of cancer, these treatments have limitations 57. It was necessary to overcome the resistance of cancer cells to HDACis and the toxic effects of HDACis. Therefore, an urgent need for HDACis with high efficiency, low toxicity and high selectivity remains. Our research screened a new structure of HDACis (Z31216525) using a combination of computer-aided drug design and experiments.

The main functional group of benzamide HDACis that chelates Zn in the active center of HDAC is the benzene ring, which is deep in the active pocket. Z31216525 has a new structure, and the chelating effect of Zn is pyridine. The N atom replaces the third C atom on the benzene ring, which also changes the space structure of the benzene ring. The ability of the benzene ring to chelate Zn in our study was weaker than that of pyridine. The reason may be that the reason may be that the alkaline of the pyridine functional group is stronger than that of the benzene ring, and because the presence of N atoms formed stronger chelates with zinc ions. This change has important value for the development of HDACis with new structures.

Z31216525 selectively inhibited the proliferation of EOC cells (A2780 and Skov3) and was less toxic to normal cells. Research data show that the antiproliferative effect of HDACis on cancer cells is primarily achieved via the induction of tumor cell cycle arrest and apoptosis 58. Z31216525 selectively induced G1- phase arrest of ovarian cancer cells and induced apoptosis of ovarian cancer cells via the mitochondrial pathway in our study, which was the same mechanism noted in previous reports. Due to the weak induction of apoptosis, we focused on verifying the c-Myc-related effects on the cell cycle pathway and ribosome biogenesis in the subsequent mechanism verification steps. We also performed animal experiments to evaluate the safety and effectiveness of Z31216525 *in vivo*. The high concentration of Z31216525 *in vivo* had a stronger antitumor effect on ovarian cancer than the positive drug SAHA and had no effect on the survival of mice.

The expression of genes in the ribosome biogenesis pathway was also suppressed as a whole. The synthesis of ribosomes in cancer cells is increased to compensate for the increase in protein synthesis and to maintain unrestricted growth 59. Therefore, ribosome biogenesis is an important pathway as a promising target for cancer treatment 60. There are few reports that HDACis regulate the ribosome biogenesis pathway in tumor cells. The c-Myc gene directly regulates ribosome biogenesis 61, 62. Therefore, Z31216525 may participate in the ribosomal biogenesis pathway by inhibiting the c-Myc gene. Previous studies showed that cancer cells were more sensitive to treatments when ribosome production was inhibited 63. Decreasing the ribosome pool of cancer cells may lead to weakened metabolism and make these cells more sensitive to stress induced by other drugs. Therefore, Z31216525 may also be used in combination with other anticancer drugs to reduce the resistance of cancer cells.

In summary, we discovered that Z31216525, a new structure of HDACis, is of great significance to the study of HDACis. Z31216525 significantly inhibited the proliferation of EOC cells *in vivo* and *in vitro*, and we also performed detailed studies on its antitumor mechanism. Z31216525 had lower toxicity than existing HDACis and may improve the sensitivity of cancer cells to other anticancer drugs. Z31216525 has great prospects in the treatment of EOC alone or in combination with other antitumor drugs.

## Supplementary Material

Supplementary figures and tables.

## Figures and Tables

**Table 1:** The IC50 values for Z31216525, Z46582199, Z165155756 and Z234820564 in different cancer cell lines and the IC10 value for HOSEpiC, the value is average and the standard deviation

Chemicals	IC10 (μM)	IC50 (μM)						
	HOSEpiC	A2780	Skov3	HepG2	Bel7402	HeLa	SW480	SGC7901
Z31216525	87.55±3.24	59.98±7.40	64.12±5.22	68.52±5.28	59.42±4.18	87.52±5.7	112±22.2	153.739±16
Z46582199	13.69±0.81	54.92±6.22	62.36±4.38	72.29±7.54	75.28±5.23	90.15±2.93	95.26±7.32	>300
Z165155756	>300							
Z234820564	>300							

**Table 2:** Changes in body weight of mice before and after treatment

Group	Before treatment (g) ± SD	After treatment (g) ± SD	Average weight gain (g)
DMSO	19.96±1.06	21.57±0.83	1.45
Z31216525 (25 mg/kg)	20.33±0.70	21.35±1.55	1.17
Z31216525 (50 mg/kg)	19.94±0.47	20.28±1.73	0.76
Z31216525 (100 mg/kg)	19.68±0.98	20.38±1.86	1.16
SAHA (50 mg/kg)	20.24±1.61	20.38±1.88	0.86
