# Supplementary material for: Discovery of a novel HDACi structure that inhibits the proliferation of ovarian cancer cells in vivo and in vitro
Source: Int J Biol Sci. 2021 Aug 12;17(13):3493–507. doi: 10.7150/ijbs.62339 (PMC8416734; doi:10.7150/ijbs.62339)
Supplement: Supplementary file 1 — Supplementary figures and tables. [file ijbsv17p3493s1.pdf]

Table S1

Gene Name	Sequences of the gene-specific primer pairs
CDKN1A(Human)	5’-GATGGAACTTCGACTTTGTCAC-3’ (forward)
	5’-GTCCACATGGTCTTCCTCTG-3’ (reverse)
DHRS2(Human)	5’-ATCCTAAGTGTGAACGTGAAGT-3’(forward)
	5’-CGCCACTACTGGATTATAAGCT-3’ (reverse)
PNRC1(Human)	5’-GCAGGATTCTGTTTCATCTGAC-3’ (forward)
	5’-ATTTTCAACAGTGCTTCCCATC-3’ (reverse)
BMF(Human)	5’-CCACCAGCCAGGAAGACAAAGC-3’ (forward)
	5’-GAGGAAGCCGATAGCCAGCATTG-3’ (reverse)
HDAC7(Human)	5’-CTCAAACTGGACAACGGGAAG-3’ (forward)
	5’-AATGAAGCTCATTCCAGATGGT-3’ (reverse)
c-Myc(Human)	5’-CGACGAGACCTTCATCAAAAAC-3’ (forward)
	5’-CTTCTCTGAGACGAGCTTGG-3’ (reverse)
CCND2(Human)	5’-TTTAAGTTTGCCATGTACCCAC-3’ (forward)
	5’-ACGTCTGTGTTGGTGATCTTAG-3’ (reverse)
BOP1(Human)	5’-TACAACCCACCCCCTGAATA-3’ (forward)
	5’-CTACATTCACCCTCATCTTGCG-3’ (reverse)

S1

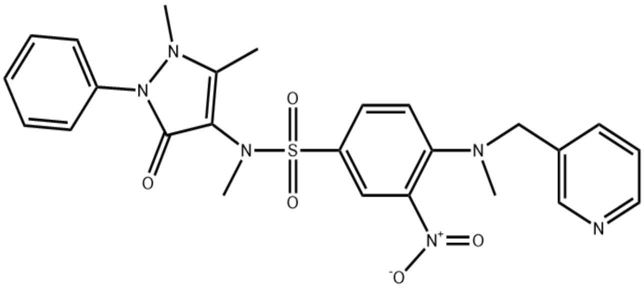

Z31216525

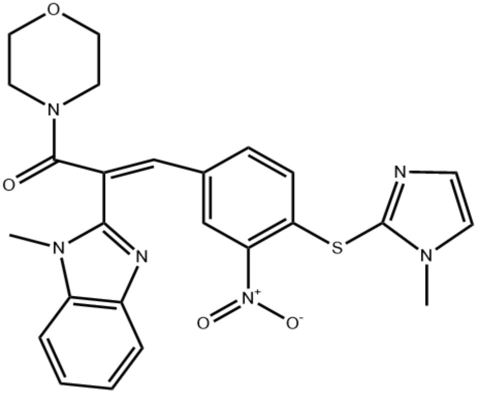

Z46582199

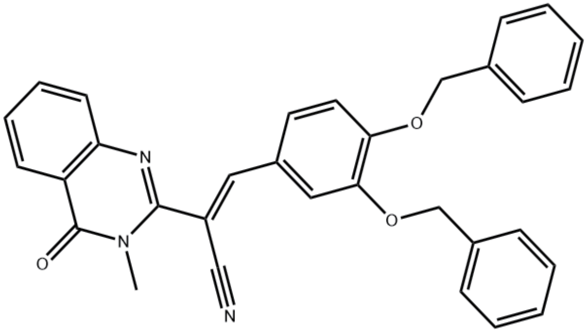

Z165155756

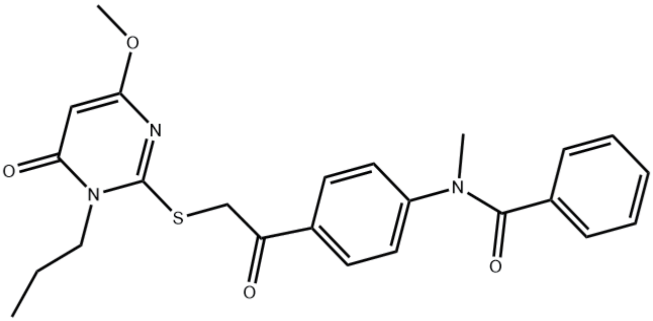

Z234820564

S2

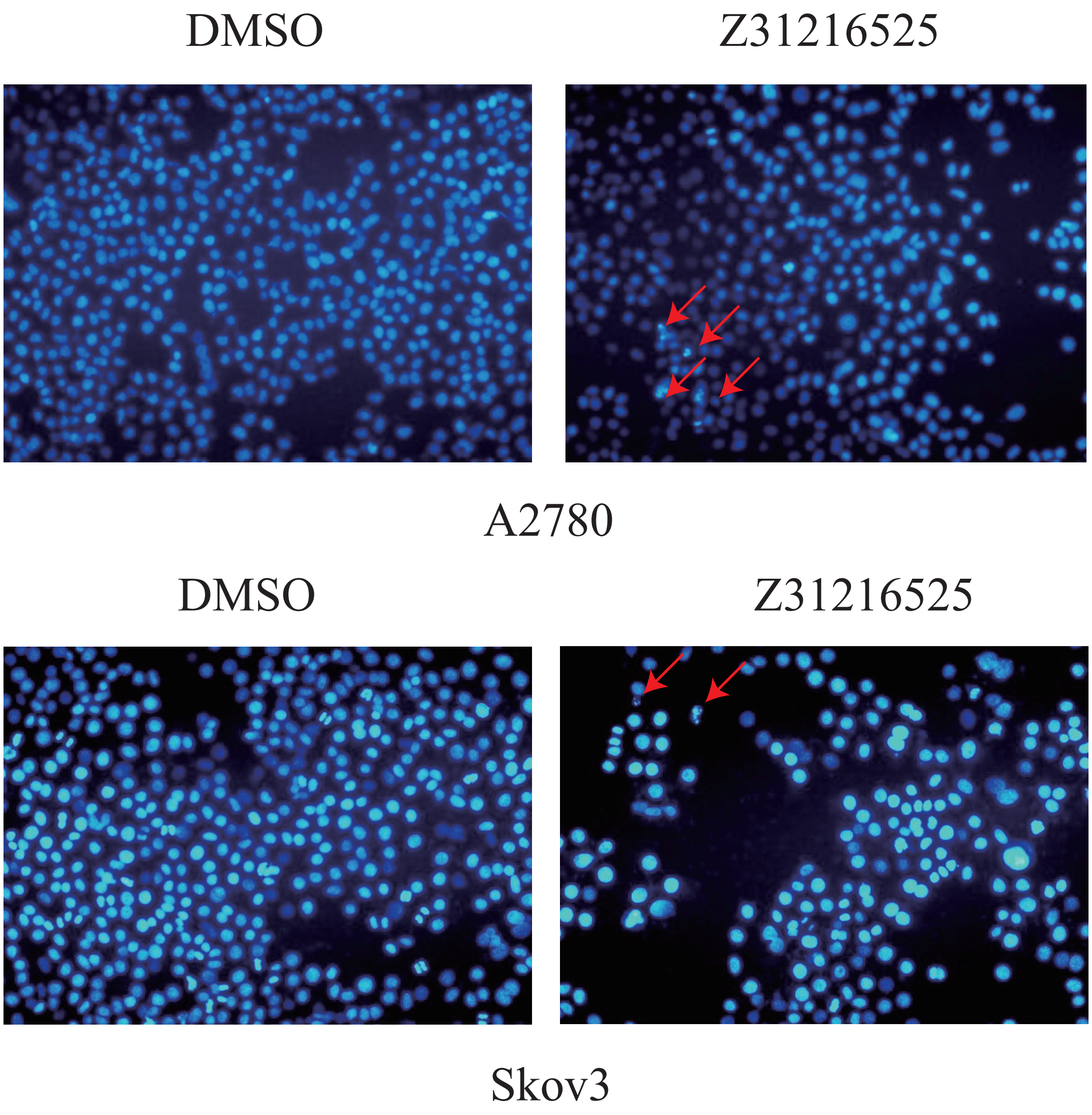

S3

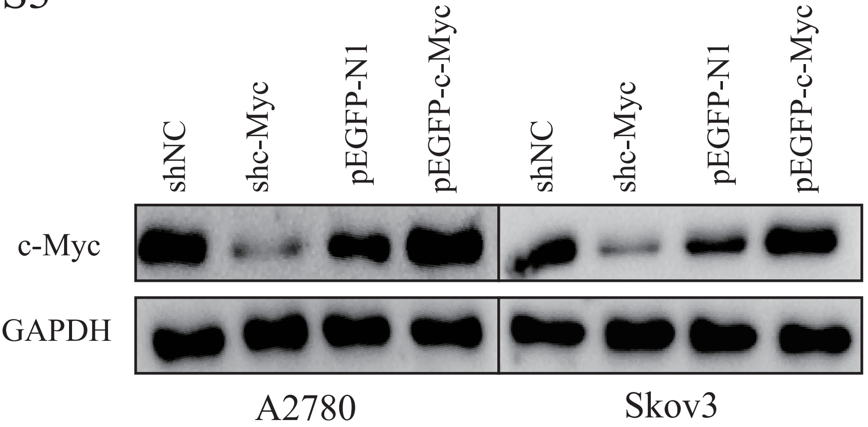
shNC
shc-Myc
pEGFP-N1
pEGFP-c-Myc
shNC
shc-Myc
pEGFP-N1
pEGFP-c-Myc
c-Myc
GAPDH
A2780
Skov3
